# Gut Microbiota Mediate *Plutella xylostella* Susceptibility to Bt Cry1Ac Protoxin and Exopolysaccharides

**DOI:** 10.3390/ijms25158483

**Published:** 2024-08-03

**Authors:** Meiling Wang, Lili Geng, Jinxi Zhou, Ziqiong Gu, Bai Xue, Changlong Shu, Jie Zhang

**Affiliations:** 1 Shanxi Key Laboratory of Integrated Pest Management in Agriculture, College of Plant Protection, Shanxi Agricultural University, Taigu 030801, China; meilingw_123@sxau.edu.cn (M.W.);; 2 State Key Laboratory for Biology of Plant Diseases and Insect Pests, Institute of Plant Protection, Chinese Academy of Agricultural Sciences, Beijing 100193, China; genglili@caas.cn (L.G.);

**Keywords:** exopolysaccharide, intestinal bacterial communities, Cry1Ac protoxin, *Plutella xylostella*, 16S rRNA

## Abstract

Exopolysaccharides (EPSs) are carbohydrate polymers that are synthesized and secreted into the extracellular during the growth of microorganisms. *Bacillus thuringiensis* (Bt) is a type of entomopathogenic bacterium, that produces various insecticidal proteins and EPSs. In our previous study, the EPSs produced by Bt strains were first found to enhance the toxicity of insecticidal crystal proteins against *Plutella xylostella*. However, the response of the intestinal bacterial communities of *P. xylostella* under the action of EPSs is still unelucidated. In this study, 16S rRNA amplicon sequencing was used to characterize the intestinal bacterial communities in *P. xylostella* treated with EPSs alone, Cry1Ac protoxin alone, and both the Cry1Ac protoxin and EPSs. Compared with the control group, alpha diversity indices, the Chao1 and ACE indices were significantly altered after treatment with EPSs alone, and no significant difference was observed between the groups treated with Cry1Ac protoxin alone and Cry1Ac protoxin + EPSs. However, compared with the gut bacterial community feeding on Cry1Ac protoxin alone, the relative abundance of 31 genera was significantly changed in the group treated with Cry1Ac protoxin and EPSs. The intestinal bacteria, through the oral of Cry1Ac protoxin and EPSs, significantly enhanced the toxicity of the Cry1Ac protoxin towards the axenic *P. xylostella*. In addition, the relative abundance of the 16S rRNA gene in the chloroplasts of *Brassica campestris* decreased after adding EPSs. Taken together, these results show the vital contribution of the gut microbiota to the Bt strain-killing activity, providing new insights into the mechanism of the synergistic insecticidal activity of Bt proteins and EPSs.

## 1. Introduction

The gram-positive insect-infecting pathogenic bacterium *Bacillus thuringiensis* is used to control agricultural pests globally, either as biopesticide or via expression in different transgenic crops [1,2,3]. Parasporal crystal proteins (Cry and Cyt) and vegetative insecticidal proteins (Vips) are the primary active insecticidal substances. To date, based on the identity of amino acid sequence, 78 groups (Cry1−Cry78) of Cry toxins have been identified. Among these, the Cry1, Cry2, and Cry9 toxins show strong toxicity to different lepidopteran pests [4,5,6]. To exert toxicity, the classical Bt action model showed that once ingested by the insect, the full-length Cry proteins (≈130 kDa) are processed by gut proteases and give rise to activated fragments (≈65 kDa) in the insect midgut. Then the activated fragments can bind to midgut-specific receptors of insects, leading to toxin oligomerization, the formation of cation-permeable pores, cell swelling, and eventual death of the insects [7]. *P. xylostella* (Lepidoptera, Plutellidae) is a highly destructive pest that poses a significant threat to cruciferous crops globally. This lepidopteran is difficult to control because it has developed resistance to most classes of insecticides and even to different Bt-based pesticides [8,9]. New efficient, biocontrol methods are increasingly needed. Many researchers have focused on the function of the *P. xylostella* gut microbiota [10]. Li et al. found that the interaction of insect gut microbiota and the Cry1Ac protoxin could accelerate the mortality of *Plutella xylostella* [11]. Our previous results showed that many Bt strains produce exopolysaccharides (EPSs) that increase the toxicity of the Cry1Ac protoxin toward lepidopteran insects [12]. However, the impacts of feeding EPSs on the gut bacterial communities of insects and the role of this effect in the synergistic activity of EPSs and Cry toxins remain unclear.

Bacterial EPSs are water-soluble carbohydrate polymers attached to the cell surface or coated the cells in an unbound form [13]. They can be isolated from many different bacterial genera and display great diversity in their primary structures, including molecular weights and monomer compositions [14]. The diversity of the primary structures of EPSs fulfills many different physicochemical properties and bioactivities of bacteria. For example, EPSs can protect bacteria from host defenses, improve environmental adaptation, and increase the pathogenicity [13]. The most thoroughly researched EPSs are those of lactic acid bacteria (LAB), which are probiotic bacteria found in the human gut [15,16]. The EPSs produced by LAB have many different biological activities, including antitumor activities, immunomodulatory, and anti-oxidant activities [17,18]. Moreover, LAB EPSs have been found to regulate the abundance of the gut microbiota in vitro. Hongpattarakere et al. found that EPSs from different types of *Lactobacillus* not only increased the abundance of beneficial gut bacteria (such as *Bifidobacteria*) but also inhibited the growth of non-probiotic bacteria (such as *Enterobacteriaceae*) [19] *Escherichia coli* is commonly found in the human gut and other mammals. EPSs from *E. coli* can also regulate the human gut bacterial community [20]. The impact of exopolysaccharides produced by Bt strains on the intestinal microbial community of insects, despite Bt being a recognized entomopathogenic bacterium, has not been previously documented.

Insect guts contain diverse microbial communities. The structure of the gut microbiota is variable in different insects and can change dynamically in answer to the various host diets [21]. The gut microbiota exhibits many functions for development, reproduction, and immune-based regulation of the physiological processes of a host [22,23,24]. In the interaction between intestinal pathogens and different hosts, insect gut microorganisms serve as symbiotic bacteria for the host to resist environmental stress and the development of insecticide resistance. They can also transform into potential pathogens or promote the infection of pathogens by altering the microbial community structure [25,26]. In recent years, the relationship between the insect gut microbiota and host mortality under Bt strain toxicity pressure has been increasingly examined. Some authors postulated that while different Cry toxins induce pore formation and subsequent impairment of the midgut epithelium which allows gut microorganisms to enter the hemocoel, that ultimately kills the host larvae via septicemia [27,28]. Hilbeck et al. found that gut bacteria could increase the toxicity of the Bt protein against different lepidopteran species (*Ostrinia nubilalis* and *Spodoptera littoralis*) [29]. The study of Li et al. showed that the Bt GS57 strain mediated dynamic changes of *S. exigua* gut microbiota and that the loss of these microbiota decreased the susceptibility of *S. exigua* to the toxic effects of the Bt strain [30]. And Li et al. found that gut bacteria could interact with Cry1Ac protoxin to promote death of *P. xylostella* and the gut bacterial diversity was reduced, but the bacterial load was enhanced [11]. These results indicate that the gut bacteria of insects have a pivotal function in the lethal activity of Bt strains and proteins. Then, does the synergistic effect between Bt EPSs and Cry1Ac protoxin correlate with alterations in the gut microbiota of the *P. xylostella*. Here, we used 16S rRNA gene sequencing, integrated taxonomic results, and functional analysis to evaluate the microbial community that inhabits the gut of *P. xylostella* and investigate whether the community was altered after the addition of EPSs to the Bt toxin. In addition, understanding the changes in the host gut microbial composition will provide new insights into the mechanisms of exopolysaccharide participating in Bt-killing activity.

## 2. Results

### 2.1. Illumina HiSeq Data

The gut bacterial composition of *P. xylostella* from four independent treatments, EPSs (E), EPSs and Cry1Ac protoxin (EP), Cry1Ac protoxin (P), negative control (CK), was analyzed by sequencing of the V3 and V4 variable regions of the 16S rRNA gene. A total of 1,969,615 raw reads were obtained. After filtering, 1,872,085 effective reads were generated with an average of 74,883 high-quality reads. The average length of each sample was 422 bp (Table S1). An average of 395, 606, 574, and 542 OTUs were generated from the CK, E, P, and EP samples at 97% sequence similarity (Table S1). The Shannon-Wiener curve approached a plateau, indicating that most of the gut microbial communities from the different treatments had adequate sequencing depth (Figure S1).

### 2.2. Alpha Diversity-Based Comparison in Different Treatments

**Figure 1:**
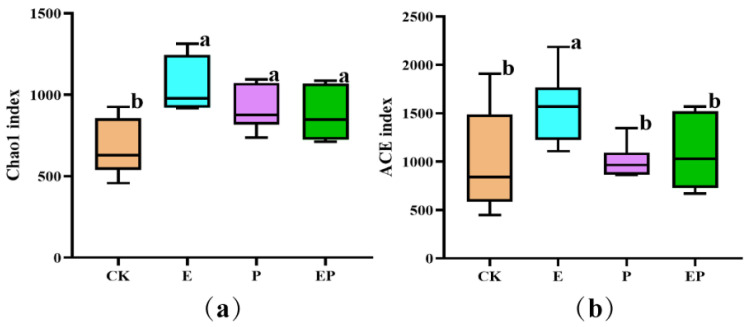
Box plots showing the distribution of alpha diversity indices of OTUs associated with gut samples of *P. xylostella* obtained after different treatments. (**a**): Chao1 index; (**b**): ACE index. CK: larvae treated with Na_2_CO_3_−NaHCO_3_ buffer; E: larvae treated with EPSs; P: larvae treated with Cry1Ac protoxin; EP: larvae treated with Cry1Ac protoxin + EPSs. The significant differences in the Chao1 index (**a**) and ACE (**b**) index were analyzed by one-way ANOVA (LSD). Different letters above the columns represent statistically significant differences between treatments (*p* < 0.05).

The alpha diversity of *P. xylostella* gut samples was assessed by four different indices (Table S2). The Chao1 and ACE indices are important for testing the number of OTUs and bacterial richness in samples. Compared with the Chao1 index value of the CK group, the values of the E, P, and EP groups were significantly higher (*p* < 0.05). Compared with the ACE index value of CK, only the E group demonstrated a significantly higher index value (*p* < 0.05). No significant differences were found in either the Chao1 or ACE indices between P and EP treatments (Figure 1). These results indicated that both the bacterial species diversity and uniformity were increased upon the addition of EPSs, whereas only the number of OTUs was increased in the P and EP groups.

### 2.3. Beta Diversity-Based Comparison in Different Treatments

**Figure 2:**
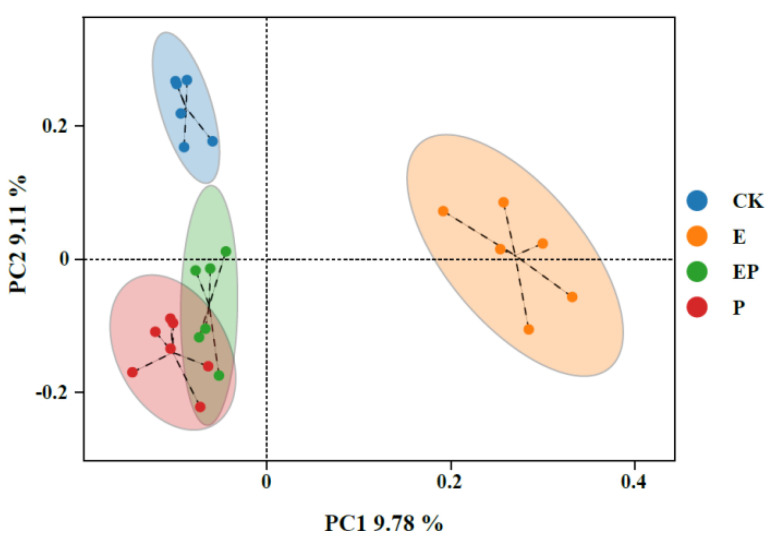
The PCoA analysis of the variance between four groups by Binary Jaccard analysis. CK: larvae treated with Na_2_CO_3_−NaHCO_3_ buffer; E: larvae treated with EPSs; P: larvae treated with Cry1Ac protoxin; EP: larvae treated with Cry1Ac protoxin + EPSs.

PCoA and ANOSIM were used to test the gut bacterial composition of *P. xylostella* following different treatments. The first principal coordinate (PC1, 9.78%) indicated that the gut bacterial communities of the E treatment group differed significantly from CK, P, and EP treatment groups. PC2 (9.11%) represented the differences between the CK group and P, EP groups (Figure 2). The ANOSIM-based effective OTUs confirmed that the E and CK groups had different bacterial compositions (R = 0.985, *p* = 0.008) (Table S3). Furthermore, the ANOSIM results showed significant differences between the P and EP groups (R = 0.765, *p* = 0.002) (Table S3).

### 2.4. Taxonomy-Based Analysis in Different Treatments

Among all treatments, 44 phyla, 398 families, and 797 genera were tested. The relative abundance and bacterial diversity of all treatments in the taxon-based analyses are shown in Tables S4–S7. Proteobacteria was the dominant phylum and contributed half of all found phylum. After adding EPSs, the relative abundances of Firmicutes (from 14.91% to 20.15%), Bacteroidetes (from 7.46% to 11.39%), and Actinobacteria (from 2.45% to 5.32%) increased, whereas those of Proteobacteria (from 56.80% to 45.80%) and Cyanobacteria (from 14.48% to 7.91%) decreased in the E group (Figure S2a). The same trend was also observed in EP groups (Figure S2a). Twenty-seven families and thirty-nine genera contributed to the differences in the E group (Wilcoxon rank-sum test, *p* < 0.05, Tables S4 and S6). Taxonomic analysis of the lower orders indicated that the enrichment of the phylum Firmicutes observed upon the addition of EPSs was caused by an increase in families, predominantly Christensenellaceae (from 0.006% to 0.282%), Marinifilaceae (from 0.039% to 0.206%) (Table S4 and Table 1). At the genus level, the relative abundance of 22 genera in the EPS group was > 0.1%. The increase in Christensenellaceae of E group was caused by a subset of genera, predominantly *Christensenellaceae_R-7_group* (from 0.005% to 0.282%), whereas the reduction of Proteobacteria was driven mainly by a decrease in *Novosphingobium* (from 0.081% to 0.001%) (Table S6 and Table 2).

**Figure 3:**
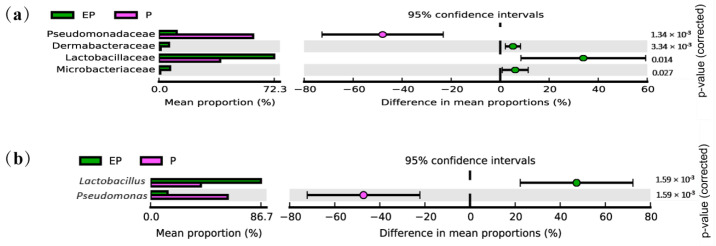
The relative abundances of families and genera differed between EP and P groups (Student’s *t*-test, *p* < 0.05). (**a**) The relative abundances of families differed between EP and P groups; (**b**) The relative abundances of genera differed between EP and P groups. P: larvae treated with Cry1Ac protoxin; EP: larvae treated with Cry1Ac protoxin + EPSs.

In total, nineteen families contributed to the differences between P and EP treatment bacterial communities (Wilcoxon rank-sum test, *p* < 0.05, Table S5). Among the top 4 different families, the increase in Firmicutes of the EP group was driven by a subset of families, predominantly Lactobacillaceae (from 0.552% to 1.638%), and the reduction in the Proteobacteria community was driven by Pseudomonadaceae (from 0.878% to 0.201%) (Figure 3a and Table S5). Compared with the P treatment, adding EPSs led to alterations in the relative abundance of 31 genera (Wilcoxon rank-sum test, *p* < 0.05, Table S7), and the relative abundance of six genera in the EP group was > 0.1%. The enrichment of Lactobacillaceae in the EP group was caused mainly by *Lactobacillus* (from 0.552% to 1.638%), whereas the reduction of Pseudomonadaceae was driven mainly by the decrease in *Pseudomonas* (from 0.878% to 0.201%) at the genus level (Figure 3b and Table S7).

### 2.5. Relative Abundance of 16S rRNA of B. campestris Chloroplast in Different Treatments

**Figure 4:**
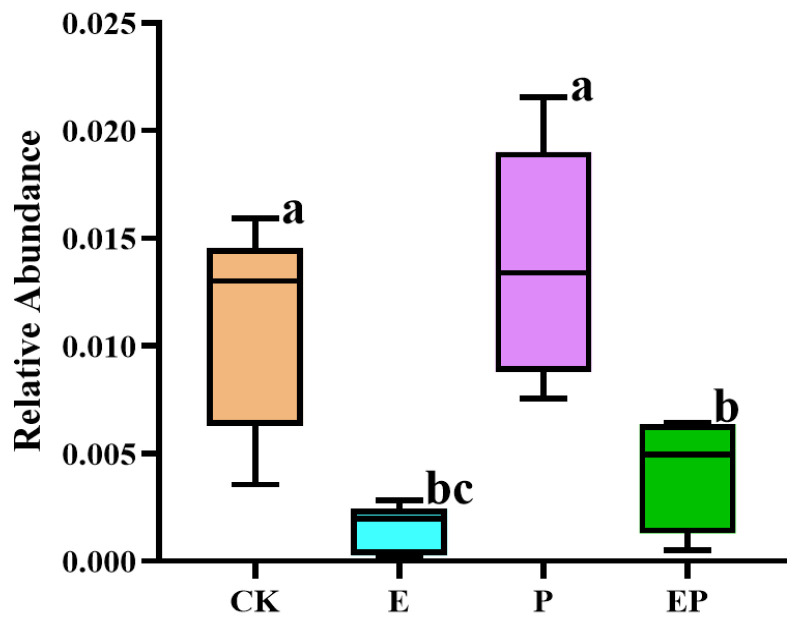
Box plots showing the relative abundance of 16S rRNA of chloroplasts of *B. campestris* in the gut of *P. xylostella*. CK: larvae treated with Na_2_CO_3_−NaHCO_3_ buffer; E: larvae treated with EPSs; P: larvae treated with Cry1Ac protoxin; EP: larvae treated with Cry1Ac protoxin + EPSs. The data were analyzed by one-way ANOVA (LSD). Different letters above the columns represent statistically significant differences between treatments (*p* < 0.05).

The 16S rRNA of the chloroplast of *B. campestris* was detected in the gut of *P. xylostella* in different treatments. Compared with CK, the relative abundance of the chloroplast 16S rRNA genes in the gut of *P. xylostella* was significantly lower in the E and EP groups. A similar variation was observed between the P and EP groups (Figure 4), suggesting that the food intake of insects in the E and EP treatment groups significantly decreased with the addition of EPSs.

### 2.6. Analysis of Co-Occurrence Network

**Figure 5:**
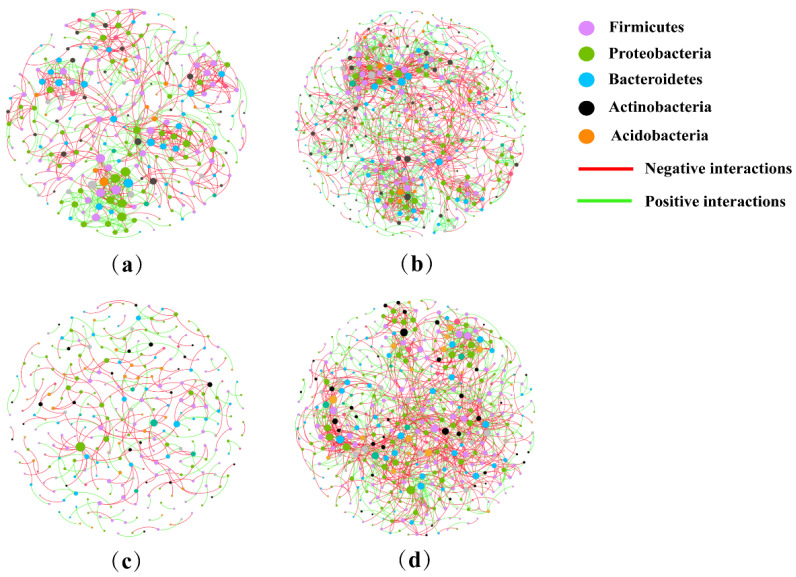
Co-occurrence networks of microbiomes of different groups. (**a**): CK; (**b**): E; (**c**): P; (**d**): EP. CK: larvae treated with Na_2_CO_3_−NaHCO_3_ buffer; E: larvae treated with EPSs; P: larvae treated with Cry1Ac protoxin; EP: larvae treated with Cry1Ac protoxin + EPSs.

The complexity of different treatment microbiomes with the addition of EPSs was evaluated by using the co-occurrence network (Figure 5). Compared to the CK, the microbiome network modularity of the EPS group showed a highly connected community with higher total nodes (495), total links (1487), greater average degree (6.008), and a shorter average path distance (5.800). Compared to the P treatment group, the EP group microbiomes showed higher total nodes (439), total links (1236), greater average degree (5.631), and a shorter average path distance (5.026). Therefore, the co-occurrence network complexity of the microbiomes was significantly enhanced by the addition of EPSs (Table 3).

### 2.7. Effect of Gut Bacteria on Axenic P. xylostella Larval Susceptibility to Cry1Ac Protoxin

**Figure 6:**
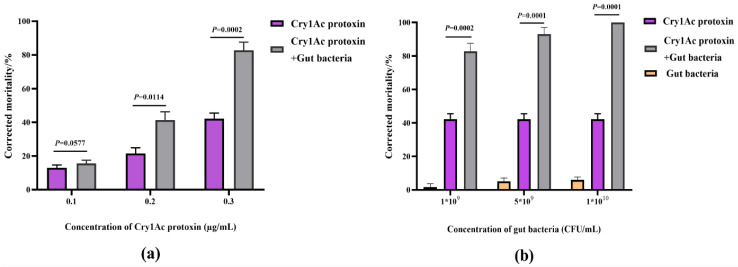
Effect of gut bacteria on *P. xylostella* larval susceptibility to Cry1Ac protoxin. (**a**): Effect of gut bacteria on the insecticidal activity of different concentrations of Cry1Ac protoxin to axenic *P. xylostella* larval (**b**): Effect of different concentrations of gut bacteria on the insecticidal activity of Cry1Ac protoxin to axenic *P. xylostella* larval. The data were analyzed by Student’s *t*-test.

To investigate the role of gut bacterial communities of insects in the synergistic activity of EPSs and Cry1Ac protoxin, axenic *P. xylostella* larvae were prepared via oral antibiotics, and gut total bacteria were isolated from the *P. xylostella* larvae feeding both EPSs and Cry1Ac protoxin. The elimination of gut microorganisms was assessed by plating the homogenates onto an LB plate, which found unculturable microbes (Figure S3). The biological activity test result showed that the corrected mortality rate of Cry1Ac protoxin to axenic *P. xylostella* was significantly increased by the addition of the gut total bacteria (1.0 × 10^9^ CFU/mL) (Figure 6a). Moreover, the synergistic effect increased with the increase of the concentration of the reintroduction gut bacteria, while the corrected mortality of different concentrations of gut total bacteria was less than 10% (Figure 6b). Therefore, gut bacteria of *P. xylostella* feeding EPSs participated in the synergistic effect of EPSs and Cry1Ac protoxin.

## 3. Discussion

The discovery of Bt strains and products based on Bt proteins generated through selective insecticidal activity has triggered great attention. To date, the crystals and spores of *B. thuringiensis* have been widely used for spray applications against different agricultural pests and pathogen vectors [31]. For an insect, the gut microbiota has been regarded as an “organ”, which plays essential roles in its health [32,33]. This coexisting relationship between insects and their gut bacterial communities is often harmonious [33,34]. However, gut bacterial communities have been implicated in opportunistic harmful interplay with their hosts. The insect immune response plays a critical role in protecting the host against infectious pathogens. Li et al. found that Cry1Ac up-regulated the *P. xylostella* midgut immune response. After removing the gut microbiota, most of the midgut immune-related genes showed down-regulated expression resulting in significantly reduced susceptibility of *P. xylostella* to Cry1Ac protoxin [11]. Elimination of gut microbiota significantly reduces the sensitivity of *S. exigua* to Bt GS57 [30]. Furthermore, a similar result was found when *O. nubilalis* and *S. littoralis* were fed genetically modified (GM) plant-produced Bt toxins [29]. These findings showed the key role of gut microbiota that mediate the insect immune response and Cry1Ac pathogenicity. Here, we tested changes in the gut microbiomes of *P. xylostella* after Cry1Ac protoxin exposure. The gut bacterial composition of *P. xylostella* in Cry1Ac protoxin-treated insects differed significantly from the control group (R = 0.88, *p* = 0.002, Table S3). At the phylum level, the findings of our study were similar to other reports in Bt-treated *S. exigua*, namely the dominant bacterial phyla, including Proteobacteria and Firmicutes. At the family level, the midgut bacteria of the Cry1Ac protoxin-treated group were mainly composed of six bacterial families: Enterobacteriaceae, Moraxellaceae, Lachnospiraceae, Flavobacteriaceae, Ruminococcaceae, and Carnobacteriaceae (Figure S2b). Our findings were similar to those of the study by Li et al., in which the dominant bacterial family was Enterobacteriaceae (Figure S2b), but their result showed the main families were Enterobacteriaceae, Enterococcaceae, Carnobacteriaceae, Rhodobacteraceae, and Mycobacteriaceae [11].

Our previous study found that 96.5% of 170 Bt standard strains cultured in the LB medium produced EPSs. Some EPSs had synergized insecticidal activity with Bt proteins [12]. The interplay between intestinal bacterial communities and *B. thuringiensis* has been investigated [11,30]; however, the effects of synergistic factors of Bt proteins, such as EPSs, on the insect gut microbiota have not been reported. In our research, we evaluated the changes in the gut microbiome of *P. xylostella* after EPS exposure using a high-throughput sequencing method. The significantly increased Chao 1 and ACE indices in EPS-treated larvae indicated that they had higher bacterial richness than the control group (Figure 1 and Table S2). The abundance of the dominant Proteobacteria was significantly reduced, whereas that of Firmicutes was significantly increased after feeding on EPSs. The analysis of the co-occurrence network suggested that EPS treatment was associated with more complex microbiomes (Figure 5). These results are the first to show that EPSs, a synergistic insecticidal factor with no insecticidal activity, can mediate significant effects on the gut microbiota of insects.

In addition, we analyzed changes in the bacterial community of *P. xylostella* after exposure to both Cry1Ac protoxin and EPSs. Compared with the Cry1Ac protoxin-treated group, the diversity and uniformity of species were not significantly altered after the addition of EPSs (Figure 1). However, PCoA, ANOSIM, and the co-occurrence network results revealed significant differences (Figure 2 and Figure 5 and Table S3). At the phylum level, the relative abundance of Firmicutes, Bacteroidetes, and Actinobacteria increased, whereas that of Proteobacteria and Cyanobacteria decreased (Figure S2a). At the genus level, the relative abundances of 31 genera were significantly different between the Cry1Ac protoxin + EPS group and the Cry1Ac protoxin-only group. We also found that the 16S rRNA relative abundance of the chloroplasts of *B. campestris* decreased in the E and EP groups. This suggests that the energy in the insect body may increase, leading to a decrease in the feeding amount of *P. xylostella* after ingestion of exopolysaccharides. However, the weight inhibition rate in the EP treatment group was higher than that in the P treatment group even with reduced feeding (Figure S4), which further indicated that exopolysaccharides played an important role.

*Trueperella* and *Brachybacteriumcan* cause serious infections in the blood and lungs of animals, and *Trueperella* can be isolated from samples of animals with septicemia [35,36]. The relative abundance of *Trueperella* and *Brachybacterium* in Actinobacteria increased in the EP treatment but not in the E treatment (Table S7). Natividad et al. found that *Bilophila wadsworthia* (belonging to *Bilophila*) induced metabolic dysfunction in mice [37]. *Pseudomonas* is a highly versatile genus, and different species can act as beneficial to plants or insect-pathogenic roles [38,39]. However, the relative abundances of *Bilophila* and *Pseudomonas* in Proteobacteria decreased after the addition of EPSs (Table S7). Therefore, we speculated that the regulation of the synergistic effect of exopolysaccharide and Bt protein by intestinal bacteria was not the result of any single bacterium genus, but the result of the action of the complex microbial community. Therefore, we isolated the total culturable intestinal bacteria after feeding exopolysaccharide and Cry1Ac protoxin and analyzed their synergistic effect with Cry1Ac protoxin. The results showed that it had a significant synergistic effect with Cry1Ac protoxin (Figure 6). It had a certain concentration dependence, which is consistent with the synergistic relationship between EPSs and Bt proteins that we found earlier [12]. These results suggested that intestinal bacteria played an important role in the synergistic insecticidal activity of EPSs and Bt proteins.

## 4. Materials and Methods

### 4.1. Insect Rearing and Gut Sampling Preparation

*P. xylostella* were initially collected from Langfang, China, and reared continuously in an insecticide-free laboratory as described by Wang et al. [40]. Second-instar *P. xylostella* larvae used for this study were fed *B. campestris* leaf discs. In order to ensure the number of samples in different treatments, *B. campestris* leaf discs were pretreated with the weight inhibition concentration of Cry1Ac protoxin (0.1 µg/mL, P), EPSs (0.5 mg/mL, E), and Cry1Ac protoxin (0.1 µg/mL) + EPSs (0.5 mg/mL) (EP). Larvae treated with Na_2_CO_3_−NaHCO_3_ buffer (25 mM) were used as the control (CK). Extraction and purification of the Cry1Ac protoxin and EPSs were conducted using the methods reported by Zhou et al. [41] and Wang et al. [12] without modifications. A total of 25 samples were obtained for the four groups, including six E samples, six CK samples, six P samples, and seven EP samples, each containing 40 *P. xylostella* larvae. The changes in weight changes in *P. xylostella* of different treatments were calculated after 72 h (Figure S4). All larval surfaces were dipped in 75% ethanol for two minutes and rinsed three times with sterile water, followed by dissection in sterile saline to obtain the guts.

### 4.2. DNA Extraction

For the extraction of gut bacterial genomes, guts from 25 samples of *P. xylostella* were extracted using the Fast-DNA Kit (Axygen Biosciences, Union City, CA, USA) according to the instructions with minor modifications. The tubes containing the gut samples and silica sand with a volume of 2.0 mL were shaken, and guanidinium isothiocyanate solution (700 µL) was added. Subsequently, the tubes were placed into the tissue disruptor and crushed for 45 s. After the agarose gel electrophoresis testing, the genomes of the gut bacteria were stored for later use.

### 4.3. 16S rRNA Gene Sequencing

Based on the Illumina HiSeq sequencing technology, the method of paired-end sequencing was used to test microbial diversity. The pair of universal primers with barcodes (primer sequences in Table S8) were used to amplify the V3 and V4 regions of the 16S rRNA tags (434 bp). All polymerase chain reactions (PCR) were performed using the High-Fidelity PCR Master Mix (Thermo Fisher Scientific, Waltham, USA), after which all products were further treated (mainly contained purification, quantification, and homogenization) to form a sequencing library. Then qualified libraries were sequenced with an Illumina HiSeq 2500 at Biomarker Inc. (Beijing, China). The sequencing results of 25 samples were deposited in the Sequence Read Archive (SRA) database (BioProject ID PRJNA1014569). SRR25990343 to SRR25990367 were the accession numbers.

Raw reads were filtered using the Trimmomatic software (version 0.33), and primer sequences were recognized and removed by Cutadapt 1.9.1. Clean reads were obtained using FLASH (version 1.2.7), which merges the reads through overlap. Effective reads were obtained using UCHIME (version 4.2) to detect and remove the chimeric sequences. Sequences were classified into operational taxonomic units (OTUs) with 97% similarity by using USEARCH (version 9.2.64). Based on the Greengenes database (version 13.8), the representative sequences were grouped into different species using the Ribosomal Database Project classifier (version 2.2).

### 4.4. Analysis of 16S rRNA Sequencing Results

The QIIME 1.9 software was used to evaluate the alpha diversity of the four groups, and all data were processed by one-way analysis of variance (ANOVA) with SPSS (version 22). Based on the Binary Jaccard distance, principal coordinate analysis (PCoA) and ANOSIM were assessed by the R software (version 2.15.3). The abundance differences between different treatments (CK and E, P, and EP) were analyzed in BMKCloud (http://www.biocloud.net/, 1 June 2024) using the Wilcoxon rank sum test. The co-occurrence network of the four groups was constructed using online analytical software (http://ieg4.rccc.ou.edu/mena/, 1 May 2024) [42], and Gephi (version 0.9.7) was used to export the network diagrams [43].

### 4.5. Isolation of Gut Microorganisms of P. xylostella

Second-instar *P. xylostella* larvae used for this study were fed *B. campestris* leaf discs, which were pretreated with Cry1Ac protoxin (0.2 µg/mL) + EPSs (0.5 mg/mL). From the surviving *P. xylostella* larvae, fifteen larvae were randomly selected for isolation of gut microorganisms after 72 h. All larval surfaces were dipped in 75% ethanol for two minutes and rinsed three times with sterile water. The *P. xylostella* guts were aseptically dissected and transferred to 100 µL axenic PBS buffer. It was then homogenized for bacterial isolation. The homogenates were plated onto the LB agar plate and incubated at 30℃ overnight. After this, the bacteria were counted by the colony counting method for further study.

### 4.6. The Preparation of Axenic P. xylostella

The fresh *B. campestris* leaves were washed with distilled water, dried in the air, and cut into circular pieces measuring 6 cm in size. The leaf discs were pierced with a sterile toothpick and dipped in an antibiotic solution containing, 75 µg/mL kanamycin sulfate, 75 µg/mL carbenicillin, 18.75 µg/mL streptomycin, 37.5 µg/mL rifampicin for 2 h. The control leaf discs were dipped in ddH_2_O. The above leaf discs were then placed in the disposable Petri dished (9.0 cm) after drying, and several newly hatched *P. xylostella* larvae were gently placed into each plate. From the surviving *P. xylostella* larvae, ten larvae were randomly selected and assessed for the efficacy of elimination of gut bacteria after 24 h. The left surviving larvae were used for subsequent bioactivity determination.

### 4.7. The Role of Gut Microorganisms in the Insecticidal Activity of Cry1Ac Protoxin

To reintroduce the gut microorganisms by oral feeding, a total of 100 µL of the total intestinal bacteria obtained in part 4.5 were spread on an LB agar plate (15 cm). After 24 h, all colonies on the plates were collected, resuspended in 5.0 mL of sterile PBS buffer, and then counted using the colony counting method for subsequent experiments. Then, the isolated gut bacteria, Cry1Ac protoxin, and the mixture of gut bacteria and Cry1Ac protoxin were fed to axenic *P. xylostella* larvae, respectively. The number of dead larvae was counted in 72 h, and the corrected mortality was calculated.

## 5. Conclusions

In summary, our results are the first to show that the bacterial community structure of *P. xylostella* was significantly changed by the addition of EPSs in both the control and Cry1Ac protoxin-treated groups. The intestinal bacteria via oral Cry1Ac protoxin and EPSs had a significant synergistic effect with Cry1Ac protoxin. These findings indicate that the gut bacterial community contributes to *P. xylostella* susceptibility to Bt toxin after the addition of EPSs. However, further experiments are required to resolve these novel findings.

## Figures and Tables

**Table 1:** The relative abundances of families differed between the control and EPS samples *.

Family	CK (Mean)	CK (Sd)	E (Mean)	E (Sd)	*p*-Value
Muribaculaceae	0.005589	0.008732	0.028923	0.028430	0.037373
Brevibacteriaceae	0.002469	0.002454	0.006880	0.003438	0.024975
Xanthobacteraceae	0.001177	0.002144	0.006622	0.005788	0.016309
Sedimenticolaceae	0.000774	0.001890	0.006093	0.014833	0.037373
k_Bacteria	0.001186	0.001945	0.005964	0.004955	0.024975
Alphaproteobacteria	0.000071	0.000153	0.005247	0.006150	0.006485
Christensenellaceae	0.000060	0.000045	0.002817	0.004417	0.037373
Gaiellales	0.000341	0.000569	0.002674	0.002219	0.016309
Akkermansiaceae	0.000356	0.000596	0.002365	0.002183	0.010406
Marinifilaceae	0.000392	0.000851	0.002056	0.002072	0.037373

* Only the top 10 families in abundance were shown here. CK: larvae treated with Na_2_CO_3_−NaHCO_3_ buffer; E: larvae treated with EPSs. The Wilcoxon rank−sum test was used to test the difference in relative abundance between different groups.

**Table 2:** The relative abundances of genera differed between the control and EPS samples *.

Genus	CK (Mean)	CK (Sd)	E (Mean)	E (Sd)	*p*-Value
*Muribaculaceae*	0.00557272	0.0087408	0.0288853	0.0283852	0.037373
*Brevibacterium*	0.002468638	0.0024539	0.00688	0.0034382	0.0249747
*k_Bacteria*	0.001186131	0.0019448	0.0059637	0.0049554	0.0249747
*Sedimenticola*	0.000543375	0.0013241	0.0058379	0.0142074	0.037373
*Alphaproteobacteria*	0.0000707	0.000153	0.0052466	0.0061503	0.0064853
*Ralstonia*	0.00000236	0.00000577	0.0043674	0.0085537	0.0039478
*Xanthobacteraceae*	0.000847237	0.0018564	0.0038452	0.0025643	0.037373
*Coprostanoligenes_group*	0.000803038	0.0013656	0.0031622	0.0017596	0.037373
*Christensenellaceae_R-7_group*	0.0000485	0.0000315	0.0028147	0.0044185	0.037373
*Desulfobacteraceae*	0	0	0.0026747	0.0065158	0.0163092

* Only the top 10 genera in abundance were shown here. CK: larvae treated with Na_2_CO_3_−NaHCO_3_ buffer; E: larvae treated with EPSs. The Wilcoxon rank−sum test was used to test the difference in relative abundance between different groups.

**Table 3:** Topological properties of different treatment networks.

Network Properties	CK	E	P	EP
Total nodes	287	495	291	439
Total links	750	1487	303	1236
Average path distance	6.092	5.800	5.527	5.026
Average degree	5.226	6.008	2.082	5.631
Average clustering coefficient	0.291	0.321	0.109	0.242

## Data Availability

The data presented in this study are openly available in the Sequence Read Archive (SRA) database (BioProject ID PRJNA1014569), reference number [SRR25990343-SRR25990367].

## Supplementary Materials

The following supporting information can be downloaded at: https://www.mdpi.com/article/10.3390/ijms25158483/s1.
