# Supplementary material for: Gut Microbiota Mediate Plutella xylostella Susceptibility to Bt Cry1Ac Protoxin and Exopolysaccharides
Source: Int J Mol Sci. 2024 Aug 3;25(15):8483. doi: 10.3390/ijms25158483 (PMC11313015; doi:10.3390/ijms25158483)
Supplement: Supplementary file 1 [file ijms-25-08483-s001.zip › Figure S.pdf]

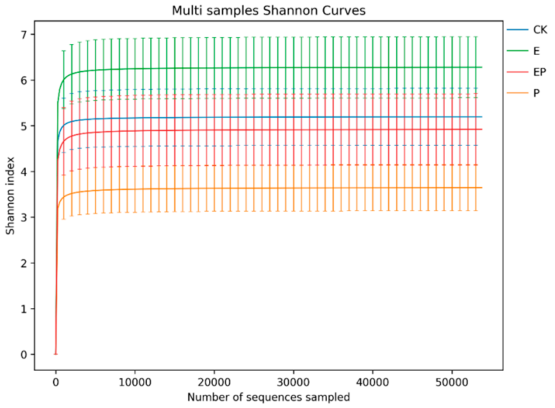


**Figure S1** Rarefaction on Shannon index of gut of *P. xylostella* from four groups, CK (n = 6), E (n = 6), EP (n = 6) and P (n = 7).

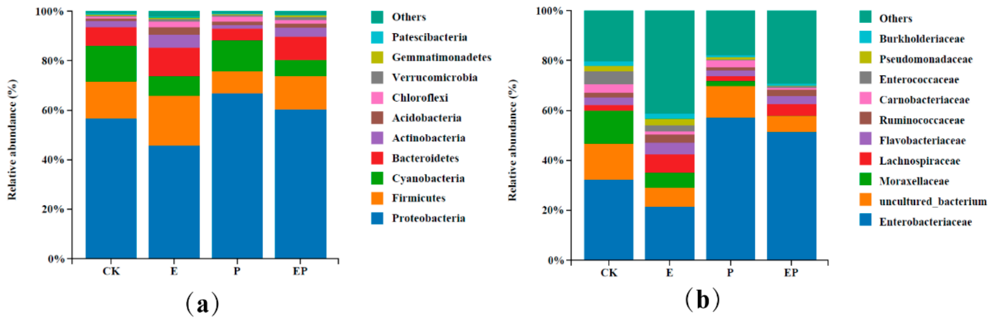


**Figure S2.** Histograms indicating the taxa-based comparisons between different treatments. (a) phylum level; (b) family level. CK: larvae treated with $Na_2CO_3$-$NaHCO_3$ buffer; E: larvae treated with EPSs; P: larvae treated with Cry1Ac protoxin; EP: larvae treated with Cry1Ac protoxin + EPSs.

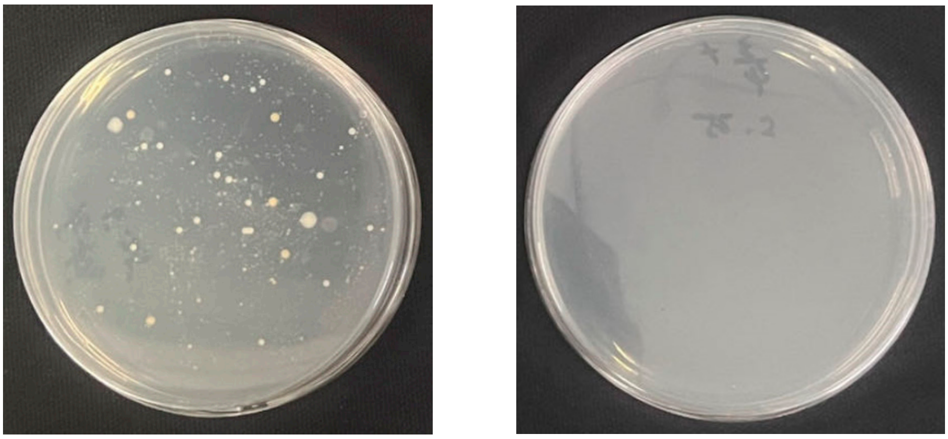

**Non-axenic** **Axenic**

**Figure S3** Generation of axenic *P. xylostella*. The efficacy of elimination of gut bacteria confirmed by culturing *P. xylostella* (n = 10) gut homogenates on LB agar plates.

In order to ensure the quantity of samples in different treatments, the weight inhibition concentration of Cry1Ac protoxin (0.1 μg/mL) and exopolysaccharides (0.5 mg/mL) were used for further analysis. The results showed that the *P. xylostella* weight of CK, E, P, EP were 0.098 ±0.007, 0.100±0.008, 0.042±0.008 and 0.0029±0.003, respectively. Compared with the control, the weight inhibition rate of the Cry1Ac protoxin treatment was 57.5% and the Cry1Ac protoxin + EPSs treatment was 70.6%, which was significantly higher than the protein-only treatment (Figure S4).

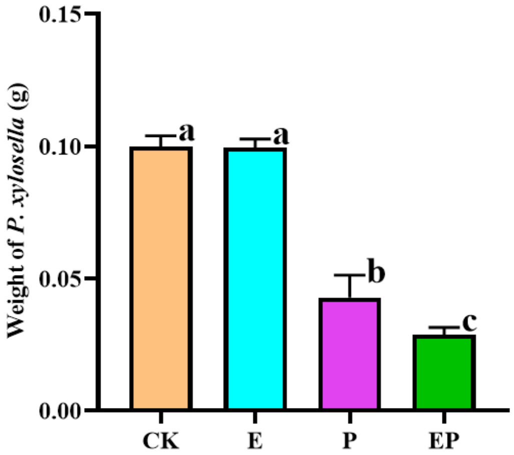


**Figure S4** Weight of *P. xylostella* from different treatments. CK: larvae treated with $Na_2CO_3$-$NaHCO_3$ buffer; E: larvae treated with EPSs; P: larvae treated with Cry1Ac protoxin; EP: larvae treated with Cry1Ac protoxin + EPSs. The significant differences of the weight in four treatments were analyzed by one-way ANOVA (LSD). Different letters above the columns represent statistically significant differences between treatments ($p < 0.05$).
